# Anti-Cancer Activity of *Buthus occitanus* Venom on Hepatocellular Carcinoma in 3D Cell Culture

**DOI:** 10.3390/molecules27072219

**Published:** 2022-03-29

**Authors:** Ayoub Lafnoune, Su-Yeon Lee, Jin-Yeong Heo, Khadija Daoudi, Bouchra Darkaoui, Salma Chakir, Rachida Cadi, Khadija Mounaji, David Shum, Haeng-Ran Seo, Naoual Oukkache

**Affiliations:** 1 Laboratoire des Venins et Toxines, Département de Recherche, Institut Pasteur du Maroc, 1, Place Louis Pasteur, Casablanca 20360, Morocco; ayoublafnoune@gmail.com (A.L.); d.khadija00@gmail.com (K.D.); darkaoui.bouchra@hotmail.com (B.D.); salmachakir19@gmail.com (S.C.); 2 Laboratoire Physiopathologie, Génétique Moléculaire & Biotechnologie, Faculté des Sciences Ain-Chock, Hassan II University of Casablanca, B.P 5366 Maarif, Casablanca 20000, Morocco; rachidacadi@gmail.com (R.C.); kh.mounaji@gmail.com (K.M.); 3 Cancer Biology Research Laboratory, Institut Pasteur Korea, 16, Daewangpangyo-ro 712 beon-gil, Bundang-gu, Seongnam-si 13488, Gyeonggi-do, Korea; suyeon.lee@ip-korea.org (S.-Y.L.); haengran.seo@ip-korea.org (H.-R.S.); 4 Screening Discovery Platform, Institut Pasteur Korea, 16, Daewangpangyo-ro 712 beon-gil, Bundang-gu, Seongnam-si 13488, Gyeonggi-do, Korea; jinyeong.heo@ip-korea.org (J.-Y.H.); david.shum@ip-korea.org (D.S.)

**Keywords:** venom, *Buthus occitanus*, hepatocellular carcinoma, multicellular tumor spheroids, anticancer activity

## Abstract

Hepatocellular carcinoma (HCC) is the most dominant primary liver cancer, which can be caused by chronic hepatitis virus infections and other environmental factors. Resection, liver transplantation, and local ablation are only a few of the highly effective and curative procedures presently accessible. However, other complementary treatments can reduce cancer treatment side effects. In this present work, we evaluated the activity of Moroccan scorpion venom *Buthus occitanus* and its fractions obtained by chromatography gel filtration against HCC cells using a 3D cell culture model. The venom was fractionated by gel filtration chromatography, each fraction and the crude venom was tested on normal hepatocytes (Fa2N-4 cells). Additionally, the fractions and the crude venom were tested on MCTSs (multicellular tumor spheroids), and this latter was generated by cultivate Huh7.5 cancer cell line with WI38 cells, LX2 cells, and human endothelial cells (HUVEC). Our results indicate that *Buthus occitanus* venom toxin has no cytotoxic effects on normal hepatocytes. Moreover, it is reported that F3 fraction could significantly inhibit the MCTS cells. Other Protein Separation Techniques (High-performance liquid chromatography) are needed in order to identify the most active molecule.

## 1. Introduction

The hepatocellular Carcinoma (HCC) accounts for more than 80% of primary liver cancer cases worldwide, mainly in developing countries in Africa and Asia [1]. HCC is an aggressive cancer that occurs as part of chronic liver disease and cirrhosis that can be diagnosed at an advanced stage [2]. It is commonly known that chronic hepatitis B and hepatitis C infections are the main causes of HCC [3]. Alcoholic cirrhosis is the second risk factor for the development of HCC in Europe and the USA [4]. In most developed countries, Non-Alcoholic Fatty Liver Disease (NAFLD) has become the most common liver disease and the major risk factor of HCC [5]. NAFLD is the cause of 10 to 20% of HCC cases in the USA [6]. Diabetes mellitus and/or obesity are known to be the main risk factors [7]. About 20% of cases diagnosed in the USA have no known predisposing risk factors (alcohol use or viral hepatitis). Strong evidence based on studies on rodent models has shed light on the role of genetic factors in the development of HCC [8]. The latter is a very complex process, involving permanent inflammatory damage that causes hepatocyte necrosis, regeneration and fibrous deposition [9]. The treatment of HCC is based on surgical methods, namely, resection, transplantation and ablation and non-surgical methods such as transarterial embolization and radiotherapy [10]. Moreover, chemical drugs such as sorafenib have also been used. Sorafenib is a multi-targeted tyrosine kinase inhibitor (TKI) with anti-angiogenic and anti-proliferative properties as well as a low toxicity profile. Sorafenib is the first drug approved as a first-line systemic treatment for patients with advanced-stage HCC that is capable of prolonging survival in patients [11].

As the current available molecules are becoming less and less effective, it is a matter of urgency to find new anticancer substances derived from natural products, alongside the discovery of new biochemical compounds with anticancer and prophylactic properties.

Nowadays, it is clear that scorpion venoms have both detrimental and beneficial effects. A significant number of studies on natural products, especially scorpion venoms, have been proven to have a variety of properties for a long time, two of which are the ability to block the growth of a wide variety of microbes and to possess immunosuppressive effects by blocking the voltage-gated potassium channel type 1.3 (Kv1.3) [12,13].

Scorpions have lived anywhere on earth for over 400 million years. Worldwide, there are 19 families of scorpions which includes more than 2200 species reported to exist so far [14]. Scorpion venoms are a complex mixture of molecules produced by the venom gland that have a very strong affinity for different target in the organisms during predatory and defensive contexts [15]. This venom is composed of a cocktail of protein substances (enzymes and peptides), non-protein substances (inorganic salts, lipids, nucleotides and amino acids) and water [16]. Despite their toxicity, scorpion venom contains numerous beneficial components, represents a promising source of compounds, which are currently being utilized for drug design in the pharmaceutical industry [17]. For these reasons, Chinese, Indian and African traditional medicines have used scorpion venoms for thousands of years to treat several diseases [18]. The major groups of scorpion venom are disulfide-bridged peptides and non-disulfide-bridged peptides and any of them have been characterized by anti-epileptic, anti-thrombotic, anti-inflammatory, anti-microbial and anticancer activities [19]. The use of scorpion venom to treat cancer has been the goal of several research teams around the world in recent years [20]. Some molecules extracted from scorpion venoms exhibit a selective cytotoxic effect in vitro against a broad type of cancer cell lines [21]. The anti-cancer activity of substances extracted from scorpion venoms has been studied in several types of cancers such as lymphoma, leukemia, glioma, neuroblastoma, cancer of the lung, breast, prostate and pancreas [22]. In vivo and in vitro studies revealed that the toxins existing in scorpion venom are able to inhibit cancer growth, induce apoptosis and limit progression and metastasis [22]. These studies have shed light on the important of applying toxins extracted from scorpion venom as novel candidate for cancer therapies [22]. Neurotoxins isolated from scorpion venom have been reported as ligands of high affinity for many ion channels. Many of their natural targets are involved in the development of cancer [23]. The anti-cancer activity of scorpion venom toxins is based on three mechanisms of action; blocking specific ion channels, activating intracellular pathways leading to cycle arrest and apoptosis and inhibiting cancer cell invasion and metastasis [24]. The anticancer properties of scorpion venom are due to its inhibitory effect on matrix metalloproteinase (MMP) activity, which leads to reduced motility and invasion in tumor cells. Venom’s inhibitory effects on MMPs also result in a decrease in the metastatic potential of malignant tumors [25,26]. However, only a few species of scorpions have been tested experimentally as anti-cancer agents [27].

The Moroccan scorpion venom *Buthus occitanus* of this study are commonly known to possess several bioactive molecules. We aimed in the first place to shed light on the chemical composition of the Moroccan scorpion venom *Buthus occitanus* and its fractions. We then tested their anticancer activity against hepatocellular carcinoma using MCTS-based phenomics screening system in a way to prove and validate the efficacy of the use of venoms in medicines. The multicellular tumor spheroid model was used by combining HCC cell line Huh7.5 together with WI38 human fibroblast cell, LX2 human hepatic stellate cell line and human endothelial cell (HUVECs). Moreover, in order to study the toxicity of venom and its fractions against normal hepatocytes, a cytotoxicity test was also carried out on Fa2N-4 cells line.

## 2. Results

### 2.1. Gel Filtration Purification of Buthus occitanus Venom

**Figure 1:**
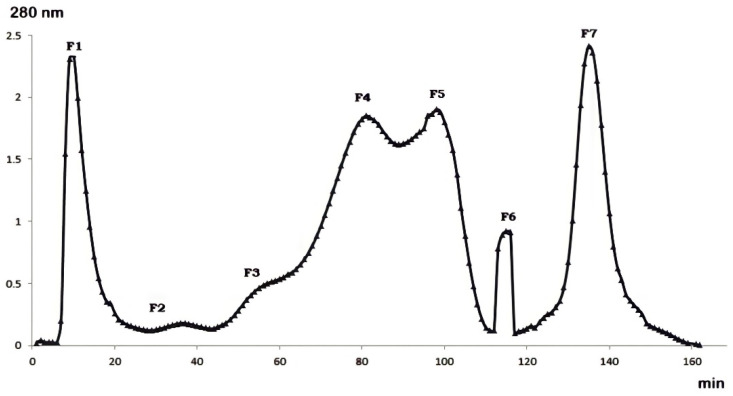
Gel filtration chromatography of *Buthus occitanus* venom on Sephadex G-50.

Fractionation of *Buthus occitanus* scorpion venom by gel filtration chromatography showed the presence of seven fractions at different concentrations (Figure 1). The seven fractions were separated by their molecular weight. F1, F4, F5 and F7 were the major fractions present in *Buthus occitanus* venom.

### 2.2. Cellular Toxicity Test against Fa2N-4 Cells (Normal Hepatocytes)

**Figure 2:**
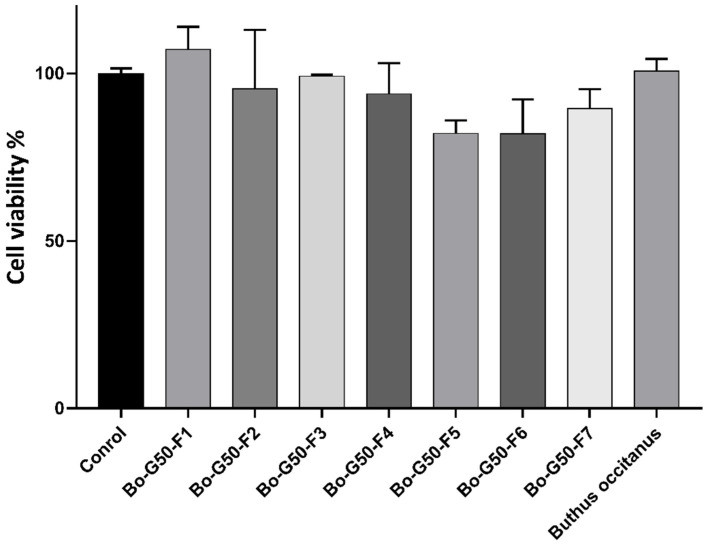
Cytotoxic activity of *Buthus occitanus* crude venom and gel filtration purified fraction on Fa2N-4 cell line. Each value is expressed as mean ± standard error of the mean (*n* = 2).

The viability histograms obtained on the Fa2N-4 cell line, after *Buthus occitanus* venom and its fractions treatment, were determined from two independent experiments. The values presented are the averages of the results obtained with their standard deviations. The cell viability is estimated as a percentage, 100% of viability corresponding to the negative control. The cytotoxicity test of *Buthus occitanus* venom and its fractions against normal Fa2N-4 hepatocytes revealed the presence of low toxicity. At concentration of 10 µg/mL, cells viability was maintained a high level (over 80%) (Figure 2).

### 2.3. Cytotoxicity Test of Venoms and Its Fractions against MCTS (Multicellular Tumor Spheroid)

**Figure 3:**
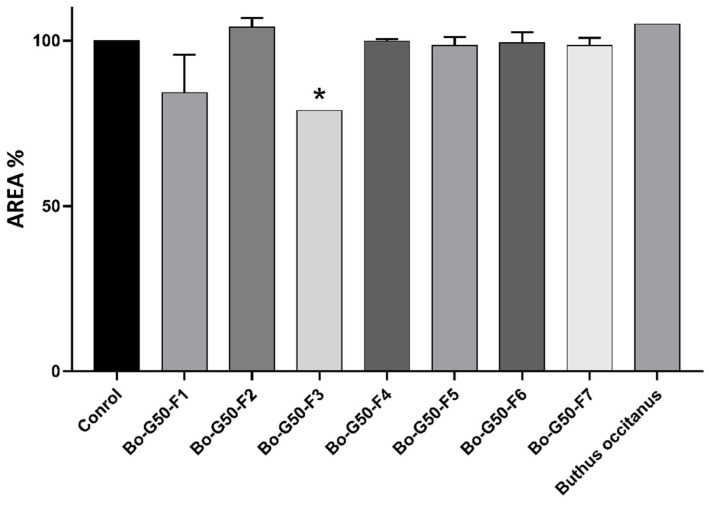
Anti-cancer activity of *Buthus occitanus* crude venom and gel filtration purified fraction on the area of the MCTSs. Each value is expressed as mean ± standard error of the mean (*n* = 2). * *p* ≤ 0.05.

At a concentration of 10 μg/mL, the 3D tumor spheroids showed a decrease of 78.91% of their cell surface after treatment with fractions F3. However, the treatment with the other fractions remained intact compared to the negative control (MCTSs treated with PBS) (Figure 3).

**Figure 4:**
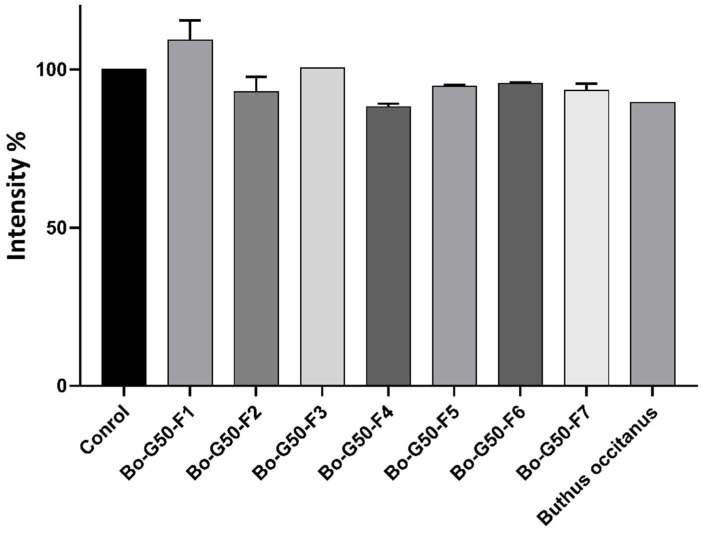
Anti-cancer activity of *Buthus occitanus* crude venom and gel filtration purified fraction on the intensity of RFP of the MCTSs. PBS was used as a negative control. Each value is expressed as mean ± standard error of the mean (*n* = 2).

The spheroids (3D culture: RFP-Huh7.5, LX2, Wi38 and HUVEC) coupled to the RFP whose utility is to mark and monitor cancer cells. The RFP is detectable in living cells, which makes it possible to measure a fluorescence emitted by RFP-Huh7.5 cells (hepato-cellular carcinoma cells). Image analysis provides access to the fluorescence intensity in the different wells. The histogram shows that, during treatment with the F4 fraction, there is a decrease in the intensity of the fluorescence at the concentration of 10 μg/mL but still statistically insignificant (Figure 4).

**Figure 5:**
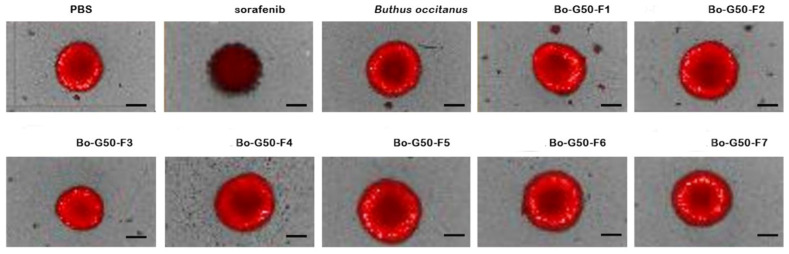
MCTSs towards *Buthus occitanus* crude venom and its fractions (10 μg/mL). Sorafenib (12.5 µM) was used as a positive control and PBS as a negative control. Scale bar = 200 μm.

Figure 5 represents the images of spheroids after seven days of incubation with a concentration of 10 μg/mL of *Buthus occitanus* scorpion venom and its seven fractions obtained by chromatography gel filtration. Moreover, it was important to note that F3 fraction decreases the spheroid size accompanied to RFP intensity. Sorafenib was used as a positive control and PBS as a negative control.

### 2.4. Characterization of F3 Fraction Using Liquid Chromatography Coupled with Tandem Mass Spectrometry (LC-MS/MS)

F3 fraction proteins were subsequently analyzed by liquid chromatography coupled with tandem mass spectrometry (LC-MS/MS). LC-MS/MS proteomic analysis revealed two proteins, namely: Alpha-insect toxin Lqq3 and Alpha-like toxin Bom4. Details on the protein characteristics and molecular function are presented in Table 1.

## 3. Discussion

Hepatocellular carcinoma is one of the most malignant diseases in the world due to its aggressive tumor biology and the lack of effective therapies. By 2025, the number of people who still be affected by liver cancer each year is estimated to be over one million [28]. Currently, surgical resection remains the standard method for treating most patients with HCC. However, the incidence and mortality rate due to HCC continue to increase rapidly caused by high rates of recurrence and metastasis after treatment [29]. Today, sorafenib is the only systemic drug adopted for the treatment of advanced cases of HCC. For these reasons, it is a matter of urgency to find new molecules to overcome this problem [30]. Recently, insect and scorpion venoms have been a subject of research as potential anti-cancer agents. Some in vitro and in vivo studies have shown that proteins and pep-tides isolated from scorpion venom are able to inhibit cell proliferation, induce apoptosis and inhibit metastasis [22]. There are several promising drug candidates from scorpion venom that show an anti-cancer activity on human glioma, lymphoma, breast cancer and hepatoma cells [22]. Scorpion venoms are a diverse mixture of proteins, peptides, neuro-toxins, biogenic amines, lipids, inorganic salts and mucoproteins, which can cause bio-chemical and toxicological effects and can provide good models for drug design and development [31].

In this work, we studied the anti-cancer activity of Moroccan scorpion *Buthus occitanus* venom and its fractions purified by gel filtration chromatography against hepatocellular carcinoma cells. Huh7.5 cancer cells line were cultured with WI38 human fibroblast cell, LX2 human hepatic stellate cell line, and human endothelial cell (HUVEC) in 3D culture model to form the MCTS (Multicellular Tumor Spheroids). Recently, three-dimensional (3D) cell culture technology has become a focus of research in cancer cell biology, using a variety of methods and materials in order to mimic in vivo tumor and its microenvironment. While they cannot replace in vivo investigations, they can help to bridge the gap between 2D in vitro studies and in vivo models in order to learn more about a compound or a crude extract [32,33].

The venom and its fractions were tested on Fa2N-4 cell line in order to assess the toxicity against normal hepatocytes. The results show that at a concentration of 10 µg/mL the *Buthus occitanus* venom and its fractions has no statistically significant cytotoxic effect on size reduction and decrease in RFP intensity on MCTS, except the significant effect observed with the F3 fraction. Moreover, the venom and its fractions showed no statistically significant toxicity against normal hepatocytes. In our previous work, we showed that the F7 fraction of gel filtration chromatography of snake venom *Naja haje* has a dose-dependent effect on reducing the size of HCC-MCTS and possesses the ability to decrease the RFP intensity of cancer line cells; this fraction showed a very weak cytotoxic effect against normal hepatocytes [34].

Peptide extracts from scorpion venom (PESV) have shown a number of pharmaco-logical properties that up-regulate caspase-3 and down-regulate Bcl-2 or modulate the activation of NF-kB to induce apoptosis, which inhibits various tumor cells. These PESVs are able to induce cell cycle arrest in the G0 phase that helps to inhibit the proliferation of tumor cells [35]. A study on the venom of the Iranian scorpion *Odontobuthus bidentatus* has shown that the crude venom induces apoptosis of hepatocellular carcinoma cells (HepG2 cells) via the mitochondrial pathway by inducing a change in the redox potential of cells. Moreover, the treatment of cells with 50 and 100 µg/mL of venom significantly inhibited the growth of HepG2 cells (*p* < 0.0001) [36]. In vitro and in vivo studies have shown that PESVs are able to enhance the activity of hepatic NK cells by regulating the NKG2D-MICA pathway and inducing more cytotoxic granules to kill tumor cells, this promising therapeutic strategy can be applied to treat hepatic carcinoma. PESV could improve lysis of HepG2 cells by not only enhancing NK cell cytotoxicity against tumor-bearing mice, but also restoring NK cell activity by improving expression of NKG2D. Moreover, they can inhibit tumor growth and prolong the survival time of tumor-bearing mice [37].

The spider venom *Haplopelma hainanum* has been shown to have potent effects of inhibiting the proliferation of HepG2 cells, by reducing the potential of the mitochondrial membrane, activating caspase-3 and 9 and inducing apoptotic cell death via the mitochondrial dependent pathway [38]. Another study on spider venom *Macrothele raven* showed an inhibition in a dose-dependent manner of the invasion and metastasis of cells in the renal capsule xenograft model of liver cancer. Its antitumor activity is related to the inhibition of PI3K-Akt-mTOR signaling [39]. At dose of 0.8 to 1.2 µg/mL, the venom of Medusa *Nemopilema nomurai* is capable of inhibiting the proliferation of HepG2 cells by induction of apoptosis, while showing no toxicity to hepatocyte [40].

An in vivo study on HCC rats treated with the crude cobra *Naja haje oxiana* venom causes an increase in ROS (reactive oxygen species) rate via the disruption of the liver mitochondria of mice, this disturbance can be related to either alteration, mitochondrial swelling and release of cytochrome c, which initiates apoptosis by activation of caspase 3 in HCC rats [41]. HepG2 cells treated with the *Echis pyramidum* viper venom showed a dose-dependent reduction in cell viability via cancer cell apoptosis compared to the un-treated cell control [42]. BthTX-I is a myotoxin isolated from the venom of the viper *Bothrops jararacussu*; this myotoxin revealed cytotoxic activity for the human HepG2 cell line by inducing cell death via apoptosis. BthTX-I may be able to promote cell cycle arrest at G0/G1 phase of murine tumor cells [43].

## 4. Materials and Methods

### 4.1. Cell Culture

The hepatocellular carcinoma cell line Huh7.5 was kindly provided from Charles M. Rice (Rockefeller University, New York, NY, USA), HUVECs (human umbilical vein endothelial cells), stromal cells WI38 (human fibroblasts) and LX2 (human hepatic stellate cells) were purchased from ATCC (Manassas, VA, USA). Meanwhile, Fa2N-4 cells (immortalized normal hepatocyte cell line) was obtained from Xenotech (Lenexa, KS, USA).

Huh7.5, WI38, LX2 and HUVECs cells were established as described previously [39]. All cell lines were cultivated at 37 °C in a humidified incubator of 5% CO_2_.

After cell attachment (3–6 h), serum-containing plating medium (XenoTech, Lenexa, KS, USA) was replaced with MFE serum free supporting Fa2N-4 cells (SF) medium (Xe-noTech, Lenexa, KS, USA) for maintaining Fa2N-4 cells in culture.

### 4.2. Venom Milking

The venom of *Buthus occitanus* was extracted by electrical stimulation of telson from scorpions held in captivity. Before being lyophilized and stored at −20 °C, the venom was treated by centrifugation at 15,000× *g* for 15 min at 4 °C [44].

### 4.3. Fractionation of Buthus occitanus Venom by Gel Filtration

Gel filtration was carried out in a column of 2.6 × 100 cm packed with Sephadex G-50 Medium gel. A total of 150 mg of the lyophilized *Buthus occitanus* venom was dissolved in eluant and was subjected to fractionation. Fractions of 2.5 mL were collected by means of a Frac-920 automatic fraction collector [45].

### 4.4. Cytotoxicity Assay of Buthus occitanus Venom and Its Fractions against Fa2N-4

Primary cultures of Fa2N-4 were seeded at 3.5 × 10^3^ cells per well in 384-well plates. After 24 h of incubation, 10 µg/mL of crude venom and its fractions resolubilized in Phosphate-buffered saline (PBS; Lonza, Basel, Switzerland) were incubated for 48 h. The positive control was 12.5 µM sorafenib (Santa Cruz Biotechnology, Dallas, TX, USA) and the negative control was PBS solution. The cells were fixed for 10 min at room temperature with 4% paraformaldehyde, then washed twice with PBS. The cells were stained with HOECHST 33342. To capture enough cells (>1000) for analysis, five image fields were collected from each well, starting at the center of the well. Cells were imaged with an Operetta high-content imaging system and Images were analyzed using Harmony software. All data are normalized to the negative control (untreated cells that received PBS) [34].

### 4.5. Anticancer Activity of Buthus occitanus Venom and Its Fractions against MCTS

To generate tumor spheroids, four types of cells (Huh7.5 cells, LX2 cells, WI38 cells, and HUVECs) suspended in HBM media were seeded at a density of 6 × 10^3^ cells per well. The plates were incubated for 3 days at 37 °C in a humidified atmosphere of 5% CO_2_. A total of 10 µg/mL of crude venom and its fractions were added and incubated for an additional 7 days. Positive and negative controls were 12.5 µM sorafenib (Santa Cruz Biotechnology, Dallas, TX, USA) and PBS solution (PBS; Lonza, Basel, Switzerland), respectively. All of the image, area and intensity analysis of the spheroid was performed using the HCS system and Harmony software. All data are normalized to the negative control (untreated cells that received PBS) [34].

### 4.6. Characterization of F3 Fraction Using Liquid Chromatography Coupled with Tandem Mass Spectrometry (LC-MS/MS)

Chromatographic analysis was carried out using an Agilent 1200 HPLC-Chip system. Mass spectrometric detection was carried out using a 6520 Quadrupole-Time of Flight (Q-TOF) operated in a nano-electrospray source (Agilent Technologies, Santa Clara, CA, USA). Data acquisition was carried out using the Peaks 7.5 software (Bioinformatics Solutions Inc., Waterloo, ON, Canada) against the UniProtKB/Swiss-Prot database downloaded in November 2018 from NCBI. The characterized proteins/peptides have been classified into different families on the basis of their function according to the UniProt database (https://www.uniprot.org, accessed on 23 February 2021).

The lyophilized *Buthus occitanus* fraction was suspended in 10 µL of 4 M urea in 100 mM NH4HCO3 (*v*:*v*), then mixed with 10 mM of freshly prepared dithiothreitol (DTT) in 100 mM NH4HCO3 (*v*:*v*). The solution was sonicated and flushed with nitrogen before being heated for two hours at 60 °C. Alkylation of the free SH-groups was performed by adding 55 mM of iodoacetamide (IAA) prepared in 100 mM NH4HCO3 (*v*:*v*). The samples were incubated at room temperature for 20 min, then 5 µL of DTT (30 nM) was added and incubated at 37 °C for 1 h to remove the excess of IAA. Subsequently, the reduced and alkylated fraction was digested using trypsin digestion with 1 µg of sequencing Grade Modified Trypsin (Promega, Madison, Wisconsin, USA). The enzymatic reaction was quenched by adding 5 µL of formic acid (FA) 5%. Formic acid (FA) and acetonitrile (ACN) were added to the digested fraction to 0.1% (*v*:*v*) and 5% (*v*:*v*) final concentrations.

For the online fractionation, two microliters of tryptic peptides were loaded and enriched on a 160 nL RP-C18 trap column, then separated on an analytical nano-column (150 mm × 75 µm) packed with ZORBAX SB-C18, 5 µm, 300 Å (G4240-62010; Agilent Technologies, Santa Clara, CA, USA). Peptides are eluted in a linear gradient from 3 to 80% ACN in 0.1% FA at 450 nL/min over 25 min. A tandem quadrupole time-of-flight (Q-TOF) mass spectrometer has been programmed and the mass range was set from 290 to 1700 *m*/*z* and from 59 to 1700 for the MS and MS/MS scans, respectively. The total cycle time was two seconds. In each cycle, the MS/MS fragmentation was assigned to five of the most abundant precursor ions by quadrupole isolation *m*/*z* [34].

### 4.7. Data Analysis

Data are the means ± standard deviations of the two independent experiments. Statistical test was performed using one-way ANOVA followed by Dunnett’s multiple comparisons test for parametric data in GraphPad Prism.

## 5. Conclusions

The study of the anti-cancer activity of Moroccan scorpion *Buthus occitanus* venom and its fractions purified by gel filtration chromatography against hepatocellular carcinoma using a 3D cell culture model (MCTS) showed that *Buthus occitanus* venom and its fractions have no statistically significant effect on MCTS, except a significant effect that was observed with the F3 fraction. Moreover, the venom and its fractions showed no significant toxic effect against normal hepatocytes, which makes it a safe anticancer agent. The finding suggests that the significant anticancer effects reported in F3 fraction was due to the presence of bioactive compounds. Our theory was that these bioactive compounds might exert a synergistic impact, and hence, the effect of F3 fraction can be more effective as compared to the isolated compounds, an element commonly observed in pharmacology.

## Figures and Tables

**Table 1:** Mass spectrometry analysis of the F3 fraction of *Buthus occitanus* venom.

Protein Name	Molecular Weight (Da)	UniProt ID	Molecular Function	Homology Degree %	Sequence
Alpha-insect toxin Lqq3	7334.93 ± 0.23	P01487	Sodium channel inhibitor activity	91.3	VRDAYIAKNY NCVYECFRDS YCNDLCTKNG ASSGYCQWAG KYGNACWCYA LPDNVPIRVP GKCH
Alpha-like toxin Bom4	7287.96 ± 0.37	P59354	Sodium channel inhibitor activity	97.7	GRDAYIAQPE NCVYECAKNS YCNDLCTKNG AKSGYCQWLG KYGNACWCED LPDNVPIRIP GKCHF

## Data Availability

Not applicable.

## Sample Availability

The Samples that support the findings of this study are available from the corresponding author upon reasonable request.
